# Measuring patients’ medical treatment preferences in advance care planning: development and validation of the Treat-Me-ACP instrument – a secondary analysis of a cluster-randomized controlled trial

**DOI:** 10.1186/s12904-024-01404-8

**Published:** 2024-03-21

**Authors:** Julia Jaschke, Rieke Schnakenberg, Katharina Silies, Almuth Berg, Änne Kirchner, Falk Hoffmann, Gabriele Meyer, Sascha Köpke, Juliane Köberlein-Neu

**Affiliations:** 1 Center for Health Economics and Health Services Research, Schumpeter School of Business and Economics, University of Wuppertal, Wuppertal, Germany; 2 Department of Health Services Research, Carl von Ossietzky University Oldenburg, Oldenburg, Germany; 3 Institute for Social Medicine and Epidemiology, University of Lübeck, Lübeck, Germany; 4 Martin Luther University Halle-Wittenberg, Halle (Saale), Germany; 5 Medical Faculty, University of Cologne, Cologne, Germany; 6 School of Business and Economics Center for Health Economics and Health Services Research, University of Wuppertal, D-42119 Wuppertal, Germany

**Keywords:** Advance care planning, Patient preference, Hypothetical scenario, Community-dwelling older person, Independent living

## Abstract

**Background:**

Advance Care Planning interventions should be evaluated as broadly as possible to gain a holistic understanding of the Advance Care Planning process. However, validated early stage outcome instruments are lacking. Therefore, the Treatment-Preference-Measure-Advance Care Planning (Treat-Me-ACP) instrument was developed and validated as part of the cluster-randomized controlled trial STADPLAN (Study on Advance Care Planning in care-dependent community-dwelling older persons) to assess the effects of Advance Care Planning interventions on patients’ medical treatment preferences.

**Methods:**

The design of Treat-Me-ACP is based on the Emanuel Medical Directive and the Life Support Preferences Questionnaires. Using a multi-stage team approach a preliminary version of the Treat-Me-ACP was developed and pre-tested. The pre-tested instrument consists of one *global medical care goal*-item, five hypothetical scenarios with five hypothetical treatments, and one *how would you feel-item* within each scenario. A total of five scenario preference scores and five treatment preference scores can be formed. This version was subsequently applied to a subsample of the STADPLAN project (*n* = 80) to assess patient’s preferences at baseline (T0) and at 12-month follow-up (T2). The further validation steps were based on this subsample and included: (1) acceptance by using completion rate and frequencies of missing data, (2) internal consistency by using Cronbach’s α to test whether it was possible to create preference scores by scenario and treatment, (3) concurrent validation examining the association between the *global medical care goal*-item and the preference scores and the association between the *how would you feel*-items and the scenario preference scores, and (4) responsiveness of the instrument to changes in preferences for life-sustaining treatments by comparing preference scores from T0 to T2 between study groups.

**Results:**

Acceptance of the instrument was high. Results of concurrent validation indicate that the five scenarios represent the *global medical care goal* well. The preference scores showed an average tendency for decreasing preferences for life-sustaining treatments across all scales for the intervention group during study follow-up.

**Conclusions:**

The Treat-Me-ACP can be used to evaluate the dynamics of patients’ medical treatment preferences in Advance Care Planning. It has been validated for care-dependent community-dwelling older persons and can be used as an additional outcome measure in evaluating the effectiveness of ACP interventions.

**Trial registration:**

German Clinical Trials Register: DRKS00016886 on 04/06/2019.

**Supplementary Information:**

The online version contains supplementary material available at 10.1186/s12904-024-01404-8.

## Background

Advance Care Planning (ACP) is “a process that supports adults at any age or stage of health in understanding and sharing their personal values, life goals, and preferences regarding future medical care” [1]. The conceptualization of ACP has broadened to a “process of health behavior change” [2] that consists not only of actions (e.g. choosing a surrogate decision maker or documenting and communicating wishes to others), but also of personal reflection and awareness of the perceived value of different health states. The variety of behavioral changes means that the effect of ACP should be measured at various points in this process [3]. The ACP Outcome Framework which was developed in 2017 using the Delphi method provides an outcome structure for the evaluation of ACP at different stages [3, 4]. Even though the importance of evaluation at different stages is discussed in the framework [3, 4], research on ACP has focused mainly on long-term outcomes. Examples are the number of completed advance directives [5, 6] the use of life-sustaining treatments, number of hospitalizations, and length of stay [7]. There are currently only a few instruments for measuring early-stage outcomes [4] like the change in awareness of ACP or dynamics of the patients’ preferences. In order to be able to select appropriate outcome instruments for conducting studies, more research is therefore needed to create reliable and valid outcome measures for early stage outcomes [3]. These early measures are particularly important for the evaluation of ACP interventions, as they allow for a more in-depth explanation of the individual’s process of change. Early outcomes can be used to assess the effectiveness of ACP interventions even when long-term outcomes such as hospitalizations and length of stay cannot be observed.

In this study, we aimed to develop and validate the patient-reported measure Treatment-Preference-Measure-Advance Care Planning (Treat-Me-ACP). In particular, we examined acceptance of the instrument and its internal consistency. We also focused on concurrent validation and the responsiveness of the instrument to changes in preferences for life-sustaining treatments. This measure complements the long-established effectiveness analysis of ACP interventions and will allow the study of the dynamics of patient preferences during an ACP process.

## Methods

### Study design and setting

#### Research context

The Treat-Me-ACP was developed, validated, and used in the STADPLAN project (STudy on ADvance care PLANing in care-dependent community-dwelling older persons). The purpose of the STADPLAN project was to develop an ACP intervention for elderly care-dependent people living at home and to evaluate the effects of this complex intervention in comparison to optimized usual care. The project comprised a two-arm cluster-randomized controlled trial (c-RCT) with a 12-month follow-up (German Clinical Trials Register: DRKS00016886 on 04/06/2019). The intervention was an adapted version of the patient-centered ACP program Respecting Choices® [8], and was conducted at the home care service level and the patient level [9]. Alongside effectiveness and process evaluation, a health economic evaluation of the intervention was conducted [9, 10]. Twenty-seven home care services in three German study regions (Oldenburg, Halle [Saale], and Lübeck) participated and were randomized [9, 11]. Recruitment of home care services took place from April 2019 to December 2019. First patient in was 28th May 2019, last patient out was 11th January 2021. More information about the project can be found in the study protocol [9, 10]. In addition, the development of the intervention [12] the main results [11] , and the process evaluation are published [13].

Ethical approval was obtained from the ethics committees of the Medical Faculties of the Universities of the Martin Luther University Halle-Wittenberg (no. 2019-045), the Carl von Ossietzky University Oldenburg (no. 2019-024), and the University of Lübeck, Germany (no. 19–080) in a joint approval. All methods were performed in accordance with this approval.

#### Study population

The study population of the c-RCT STADPLAN included 380 patients aged 60 years or older. The study was originally designed to include patients aged 65 years or older. The minimum age was lowered to 60 as a result of the previous pilot study, as the home care services stated that it was important not to exclude “younger” patients. Informed consent was obtained from all patients and their legal guardians. Clients of home care services who were assigned to a care grade (as assessed by expert raters of the Statutory Health Insurance) and rated to have a life expectancy of at least four weeks were included. In addition, adequate knowledge of German and the cognitive ability to follow the intervention and data collection were required. Cognitive ability to follow the intervention was assessed using the Dementia Screening Scale (DSS) [14]. Patients with a score < 3 were included in the study. Patients with a score of 3 to 5 were included if the trained nurse from the participating home care service considered the patient to be cognitively able to follow the intervention [9, 11].

This validation study used a convenience sample of 80 patients from the participants in the STADPLAN c-RCT: 40 patients of 13 home care services assigned to the intervention group (IG), 40 clients of 12 home care services assigned to the control group (CG). Patients were selected by the research assistants who conducted the interviews depending on whether they felt that an additional survey using the Treat-Me-ACP could be conducted without imposing a heavy burden on the patient. The analyses of this validation study included 64–80 cases each. On average, data from 69 patients (SD = 2.2) were included in the analyses. The recruitment target of the main study was *n* = 960. The recruitment target for this validation study was 12.5% (*n* = 120) of the patients included in STADPLAN. The recruitment goal was limited by the available staff resources.

### Instrument development and pre-test

#### Step 1: development of a preliminary version

The Treat-Me-ACP was developed during the STADPLAN project. The design of the Treat-Me-ACP is based on the Life Support Preferences Questionnaires [15, 16] and the Emanuel Medical Directive [17], which were identified in a literature review. The instruments are the only ACP preference instruments known to us that have been scientifically studied. Both instruments were translated into German independently by two researchers using the multi-stage translation–review–adjudication–pre-test–documentation (TRAPD) team approach [18]. Within a project meeting, each translated element was rated by interdisciplinary research team members (RS, KS, AB, ÄK, FH, GM, SK) according to its relevance, acceptability, and appropriateness for the German healthcare context. The research team was qualified to assess the elements because of its composition of an epidemiologist, health services researchers, nursing scientists, and an nurse. To reach an agreement, at least one member of the study team from each site had to vote in favor of including the translated element. Thus, an approved item always received the approval of more than 50% of the research team members. Unlike Gilbert and Prion 2016 [19], we chose this threshold in order to include a larger number of items in the testing. Elements that received consensus were used for a preliminary version of the instrument. The result was an instrument with nine scenarios. The preliminary version was ordered by increasing intensity of health limitations and translated into patient-friendly language by experts.

#### Step 2: Assessment of comprehensibility, acceptability, and feasibility

Pre-testing of the preliminary version of the Treat-Me-ACP was conducted in two rounds of interviews with probing questions to test for comprehensibility, acceptance, and feasibility [20, 21]. The results of the interviews were evaluated and discussed within the research team and adjustments were made as needed.

#### Step 3: final interview round with adapted instrument

The final step was to test the revised instrument in interviews with probing questions to test for comprehensibility, acceptance, and feasibility again. The final adjustments were made after consultation within the research team (see Additional file 1).

#### Final instrument

**Fig. 1:**
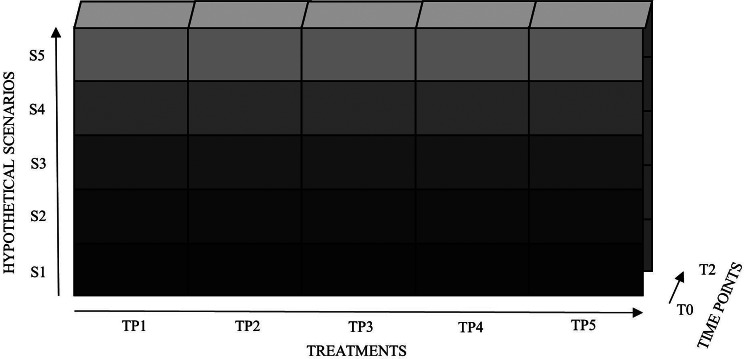
The Treat-Me-ACP instrument (modified version based on Schwartz et al. 2004 [17])

The final Treat-Me-ACP contains one *global medical care goal*-item and five scenarios: S1 (current health status), S2 (advanced dementia), S3 (stroke with paralysis), S4 (stroke with six weeks coma), S5 (incurable brain tumor) (see Additional file 2). Scenarios are roughly sorted by severity of the associated health limitations, with the most severe being the final scenario. Figure 1 shows a schematic diagram of the Treat-Me-ACP.

The Treat-Me-ACP items were used to understand the dynamics of patient preferences over the follow-up period and the differences between both study groups. We assumed that preferences could change meaningfully through an ACP intervention. Therefore, we expected to see greater changes in preference scores in the IG of the STADPLAN c-RCT than in the CG.

For each scenario, patients are asked how they would feel if they spent the rest of their lives in this state (*how would you feel*-item) and what their treatment preference (TP) would be: TP1 (antibiotics in the case of a severe infection), TP2 (cardio pulmonary resuscitation in the case of cardiac arrest), TP3 (cholecystectomy in the case of a gallbladder infection), TP4 (temporary artificial nutrition if they are unable to eat and drink independently), and TP5 (permanent artificial nutrition if they are unable to eat and drink independently). Response options were presented on a 5-point Likert scale (“definitely not” to “definitely”) supplemented by “not applicable” and “I do not wish to answer”.

The *global medical care goal*-item includes the question of whether patients prefer care or treatment that allows them to live as long as possible but may lead to health problems or care or treatment that might shorten their life but potentially reduce the risk of major health problems.

### Psychometric evaluation

#### Data collection

For psychometric evaluation of the Treat-Me-ACP, a secondary analysis of the health economic evaluation sub-study data was conducted. In addition to the Treat-Me-ACP, information was collected on patients’ sociodemographic characteristics, current health status, and use of health services in the past three months.

Data were collected at two time points of the c-RCT: T0 (baseline) and T2 (12-month follow-up). The instrument was the last part of a case report form to be completed during a structured face-to-face interview conducted by trained research assistants in patients’ homes. Each interview was scheduled individually. A family member or close friend was allowed to participate. Patients could ask comprehension questions at any time during the interviews. The research assistants trained for the STADPLAN project were qualified to answer these questions. We report methods and results of the psychometric evaluation according to the recommendations set out in Streiner et al. 2014 [22].

#### Preference scores

Following Schwartz et al. 2004 [17], two types of preference scores were created and used in this validation study: (1) preference scores across treatments within a scenario (scenario preference score, or SPS), indicating patients’ preference for a given intensity of life-sustaining treatments in the respective health status; and (2) preference scores for a specific treatment across scenarios (treatment preference score, or TPS), indicating patients’ overall preference for a given intensity of life-sustaining treatment across the five health states (see Table 1). Thus, a total of 10 preference scores per time point were formed by assigning values from 0 (= definitely not) to 4 (= definitely) to the answer choices and then adding them up. The value range of each preference score is 0 to 20, higher scores indicating higher preference for life-sustaining treatments (SPS) or higher preference for a particular treatment (TPS).

**Table 1:** Baseline patient characteristics

	Intervention group (*n*=40)	Control group (*n*=40)	Total (*N*=80)
**Age in years**			
Mean (SD)	75.98 (9.86)	78.47 (10.178)	77.22 (10.04)
Median [IQR]	77.50 [67.25–82.75]	79.50 [70.25–85.75]	79.00 [69.00–85.00]
**Female**	32 (80.0%)	30 (75.0%)	62 (77.5%)
**Living alone**	30 (75.0%)	29 (72.5%)	59 (73.8%)
**Long-term care grade** (*n* = 79)*			
None/1 (no or low limitations)	5 (12.5%)	9 (22.5%)	14 (17.7%)
2 (substantial limitations)	23 (57.5%)	25 (62.5%)	48 (60.8%)
3 (severe limitations)	11 (27.5%)	4 (10%)	15 (19.0%)
4/5 (very severe limitations)	0 (0.0%)	1 (2.5%)	1 (1.3%)
**ACP document completed**	32 (80%)	31 (77.5%)	63 (78.8%)
**Comorbid diagnosis, lifetime prevalence**			
Heart diseases	23 (57.5%)	19 (47.5%)	42 (52.5%)
Fractures	23 (57.5%)	24 (60.0%)	47 (58.8%)
Diabetes (*n*=79)*	10 (25.0%)	10 (25.0%)	20 (25.3%)
Cancer	10 (25.0%)	12 (30%)	22 (27.5%)
Stroke (*n*=78)*	6 (15.0%)	6(15.0%)	12 (15.4%)
Chronic obstructive pulmonary disease	8 (20.0%)	6 (15.0%)	14 (17.5%)
Parkinson’s disease	1 (2.5%)	1 (2.5%)	2 (2.5%)
Dementia	0 (0.0%)	1 (0.0%)	1 (1.3%)
**Hospitalization, last 6 months** (*n*=79)	13 (32.5%)	16 (40.0%)	29 (36.7%)

* Number of patients differs from *n*=80 because of missing values

#### Data analysis

Descriptive statistics of baseline data were calculated for the acceptance of the instrument, *global medical care goal*-item, *how would you feel*-items, and for SPS and TPS. Missing data were not imputed. Cases were excluded for the respective SPS if an item of the scenario was not completed. The same applied to the TPS when items of the respective treatment scale were not completed as well as the *global medical care goal*-item.

In accordance with other psychometric evaluations [23–25] the *acceptance* of the instrument was assessed based on data from T0 by using completion rate and frequencies of missing data per item and of the whole instrument. The response options “not applicable” and “I do not wish to answer” were also counted as missing values, since these do not provide any content-related information about patients’ preferences.

The *internal consistency* (Cronbach’s α) of the preference score scales was calculated based on data from T0 as in Schwartz et al. 2004 [17] to test whether it was possible to create preference scores by scenario and treatment.

For *concurrent validation* as part of the criterion validation [26], the association between the *global medical care goal*-item and the SPS, both measured at baseline, was examined using Cramér’s V as was the association between the *how would you feel*-items and the preference scores. Based on Schwartz et al. 2004 [17], it was expected that patients who preferred a longer life with possible health impairments would have higher preference scores than patients who preferred a shorter life without major health problems. By analogy, it was expected that patients who indicated on the *how would you feel*-item that living with the scenario health state would be acceptable would have a higher preference score than patients who indicated that life with the respective health state would be barely livable or not livable at all.

In addition, data from T0 and T2 were used to assess the *responsiveness of the instrument to changes in preferences for life-sustaining treatments*. For this, changes in preference scores from T0 to T2 were compared between and within study groups using non-parametric tests. On average, preference scores were expected to decrease in the IG, because preferences for life-sustaining treatments were expected to decrease. This assumption was made based on the results of systematic reviews indicating that ACP reduces preference for life-sustaining treatments [7, 27].

## Results

Baseline characteristics showed no major differences between IG and CG. The mean age of the people participating in the validation study was 77 years (SD = 10.0), and 78.5% of participants were women; 36.3% had been hospitalized at least once in the last six months, and more than 80% had at least substantial limitations in activities of daily living (care grade 2 or higher; see Table 2).

**Table 2:** Descriptive statistics of preference scores, internal consistency and concurrent validation parameters

	Preference scores				Internal consistency	Association of preference scores and global medical care goal			Association of scenario preference scores and *how would you feel*-items		
	Preference scores T0		Mean (SD) changes (T0-T2) in preference scores								
Preference score scale	n	Mean (SD) T0	IG	CG	Cronbach’s α	n	Cramér’s V	Significance	n	Cramér’s V	Significance
**Scenario**											
S1 – Current health status	69	11.6 (4.7)	-2.2 (3.4)	-1.2 (3.2)	0.718	66	0.596	0.136	69	0.805	0.103
S2 – Advanced dementia	69	5.6 (5.4)	-1.4 (4.5)	0.3 (5.4)	0.828	67	0.721	0.003	68	0.748	< 0.001
S3 – Stroke with paralysis	70	6.1 (5.6)	-2.0 (4.0)	-1.2 (5.6)	0.838	68	0.636	0.051	68	0.889	< 0.001
S4 – Stroke with six weeks coma	71	4.9 (5.6)	-1.5 (3.7)	-0.8 (4.9)	0.875	69	0.643	0.012	70	0.700	< 0.001
S5 – Incurable brain tumor	66	3.9 (4.9)	-1.2 (3.2)	-1.9 (4.8)	0.838	64	0.534	0.195	65	0.836	< 0.001
**Treatment**											
TP1– Antibiotics	70	9.9 (5.8)	-1.5 (5.3)	-0.2 (4.6)	0.812	68	0.568	0.144			
TP2 – Resuscitation	69	5.0 (5.6)	-1.4 (2.1)	-1.4 (3.7)	0.854	67	0.694	0.002			
TP3 – Cholecystec-tomy	65	9.7 (6.1)	-2.9 (6.1)	-1.7 (5.1)	0.856	63	0.617	0.198			
TP4 – Temporary artificial nutrition	72	5.7 (6.4)	-3.3 (5.3)	-1.1 (5.9)	0.918	70	0.684	0.026			
TP5 – Permanent artificial nutrition	71	1.7 (4.2)	-0.2 (2.3)	0.7 (2.1)	0.962	69	0.696	< 0.001			

Note: The minimum value of the preference scores is 0; the maximum value is 20. The higher the preference scores, the stronger the preference for life-sustaining treatments.

On average, a Treat-Me-ACP interview lasted just over 17 min (*n*=73; SD=9.0). The shortest lasted 5 min, the longest 43 min. Only interviews that contained no more than five missing values were considered for the evaluation of the interview duration.

### Descriptive analysis

#### Global medical care goal

Of all patients, 76.6% stated at T0 that they would prefer care that is more likely to shorten their lives but causes no major health problems; the rest (23.4%) preferred to receive care aimed at living as long as possible, despite higher risk of major health problems.

#### How would you feel-items

**Fig. 2:**
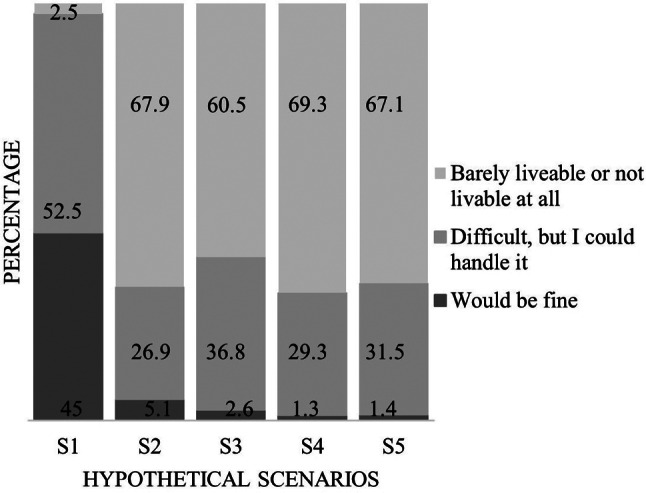
Response distribution of the *how would you feel*-items per scenario

The descriptive evaluation shows that 45% of patients would be content living with their current health status. Only 2.5% stated that life with this health status would be barely livable or not livable at all. The evaluation of the remaining items showed strong deviation from this result. More than 60% (60.5–69.3%) of participants considered life with health limitations as described in the scenarios to be barely livable or not livable at all. Only 1.3–5.1% stated that life with these limitations would be fine for them. Figure 2 shows the descriptive results of the *how would you feel*-item for each scenario.

#### Preference scores

Table 1 summarizes the descriptive statistics of the SPS and TPS across both study groups. The results demonstrate a wide range of SPS. S1 (current health status) had the highest preference scores, with a mean value of 11.58 (SD = 4.7), indicating that patients had stronger preferences for life-sustaining treatments in S1 than in S5 (incurable brain tumor), for which they stated the lowest preferences for medical care treatment (M = 3.9, SD = 4.9).

In comparison, all SPS show a tendency for less severe health limitations to lead to a higher preference for medical care treatment. Similar results were observed for the TPS. Mean TPS ranged from 9.9 (SD = 5.8) for TP1 (antibiotic treatment) to 1.7 (SD = 4.2) for TP5 (permanent artificial nutrition).

### Validation

#### Acceptance of the instrument

The average completion rate of the instrument was 93.6% (SD = 0.2). The *global medical care goal*-item was completed by 96.2% of the patients. Across all *how would you feel*-items, the mean completion rate was 95.5% (SD = 3.4).

On average, 6.5% of the answers per item and two items per patient (SD = 5.3) were missing values. The number of missing values per item differed between scenarios. The more severe the health limitations described by a scenario, the higher the number of missing values. For example, items of S1 had on average 3.5% (SD = 3.3) missing values, for S5 the average of missing values was 11.3% (SD = 1.8). More information on the missing values can be found in Additional file 3.

#### Internal consistency

Cronbach’s α shows high values for nine out of 10 scales (0.812–0.962; see Table 1). The scenario of current health status has a slightly lower but still sufficient value of 0.718. Based on Cronbach’s α for the scenarios, we conclude that patients made consistent decisions for or against life-sustaining treatments within a scenario and thus that calculating the SPS as intended is possible. Cronbach’s α of the treatment scales shows consistent decisions for or against a treatment across the different scenarios, and so the calculation of the TPS is also possible as intended.

#### Concurrent validation

**Fig. 3:**
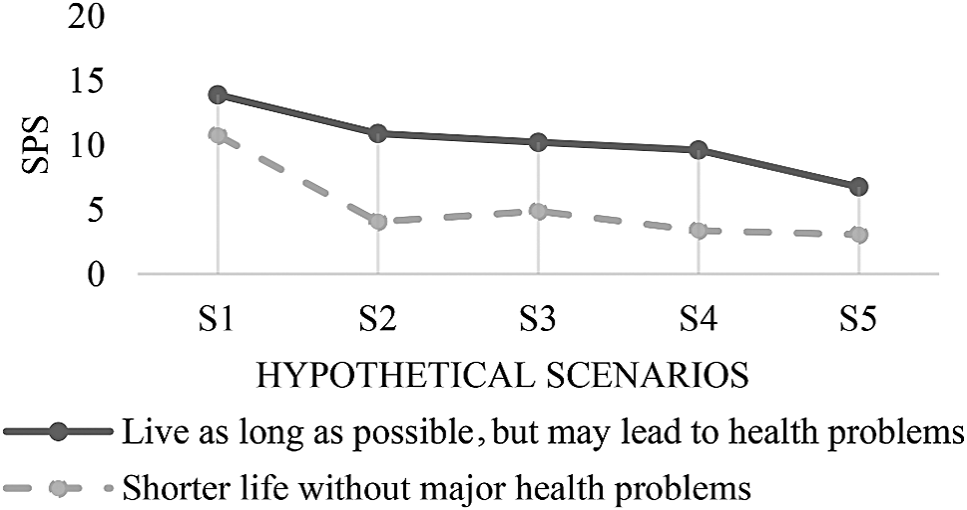
Association of the global medical and home care goal and the scenario preference scores

**Fig. 4:**
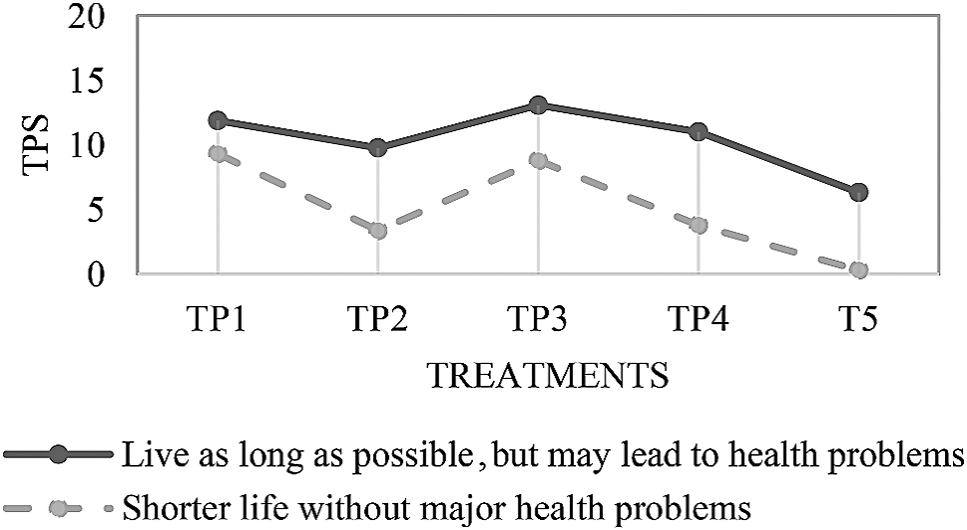
Association of the *global medical and home care goal*-item and the treatment preference scores

For concurrent validation, the association between two criteria that were collected at almost the same time is analyzed [26]. Patients who indicated on the *global medical care goal*-item that they preferred a longer life with possible health limitations had consistently higher preference scores than patients who preferred a shorter life without health limitations (see Figs. 3 and 4). Cramér’s V shows strong associations between the *global medical care goal*-item and all preference scores (see Table 1). The association is statistically significant for S2 – Dementia, S4 – Stroke with six weeks coma, TP2 (resuscitation), TP4 (temporary artificial nutrition), and TP5 (permanent artificial nutrition).

**Fig. 5:**
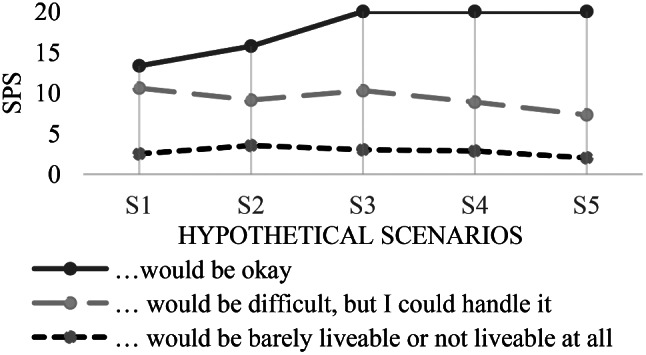
Association of the *how would you feel*-items and the scenario preference scores

Patients who indicated that living with the respective health status would be acceptable had on average the highest preference scores. Patients who stated that such a life would be barely livable or not livable at all had on average the lowest preference scores for all scales (see Fig. 5). Table 1 shows strong associations between the *how would you feel*-items and the SPS (statistically significant for S2 (advanced dementia), S3 (stroke with paralysis), S4 (stroke with six weeks coma), and S5 (incurable brain tumor).

#### Responsiveness of the instrument to changes in preferences for life-sustaining treatments

Baseline data show no statistically significant differences in preference scores between study groups. At T2, descriptive examination revealed a trend towards reduced preference scores for the IG across all scales, indicating a decreased preference for life-sustaining treatments in the IG (not statistically significant). In the CG, an increase in preference scores was observed for the scales S2 (advanced dementia) and S5 (permanent artificial nutrition) (see Table 1), indicating no consistent trend towards less invasive or life-sustaining treatments. Overall, with the exception of S5 (incurable brain tumor), the IG showed a greater reduction in preference scores than the CG when both groups showed a reduction.

## Discussion

In this study, we aimed to develop and validate an instrument assessing dynamics of patient preferences during an ACP process (Treat-Me-ACP). Overall, the results show good psychometric characteristics regarding acceptance and feasibility. The average number of missing values varied widely within the scenarios and increased towards the end of the instrument. There are two possible explanations for this phenomenon. Firstly, scenarios were arranged in increasing intensity, as in the Life Support Preferences Questionnaires [15, 16] and the Emanuel Medical Directive [17]. This means that scenarios involving more severe health limitations were surveyed later in the interview. Preference scores tend to be lower for the more intensive scenarios, indicating a lower preference for life-sustaining measures. The intensity of the health limitations and stress caused by the scenarios presented to the patients later may be higher than in the earlier scenarios, thus increasing the stress on the patients and the probability of not answering the items. Secondly, we assume that patients’ attention and concentration may decrease during the interview. The Treat-Me-ACP was the last part of the interview. In the study population, characterized by older age and care-dependency, patients’ ability to concentrate may have been exhausted by the end of the interviews, causing increasing numbers of missing values [28–30]. Further research should evaluate whether the order of the scenarios has an impact on the completion rate and whether the increasing number of missing values is associated with a decreasing ability to concentrate during the survey.

Cronbach’s α shows that calculating preference scores for this study population is possible by summation. In contrast to Schwartz et al. 2004 [17], no item had to be excluded from the calculation of preference scores, because we only asked about treatments that have a life-sustaining character and are not a general component of palliative care. Cronbach’s α is slightly lower for S1 (current health status) than for the other scenarios. This may be due to the fact that in contrast to the other scenarios, patients refer to their own health status when answering S1 rather than to a given description of the health status. The high Cronbach’s α may also be an indication that the scenarios and treatments can be adapted to the context of planned studies. For that, it must be ensured that (1) the scenarios differ in their accompanying health limitations and (2) the treatments differ in their intensity or invasiveness and have a life-sustaining effect (no palliative use).

Strong associations between the *how would you feel*-items and the SPS and the association between the *global medical care goal*-item and the two types of preference scores indicate satisfactory concurrent validity, suggesting that the scenarios represent the global medical care goal well. Our findings are consistent with those of Schwartz et al. 2004 [17]. A global medical care goal alone cannot adequately capture nuanced changes in wishes and preferences. Complex interventions require an in-depth understanding of their potential effects [31, 32]. The detailed portrayal of different health states and treatments by the Treat-Me-ACP thus supports appropriate evaluation of complex ACP interventions [31, 32].

As expected, the examination of *responsiveness of the instrument to changes in preferences for life-sustaining treatments* showed an average tendency for decreasing preferences for life-sustaining treatments across all scales for the IG during study follow-up. When the CG showed a reduction as well, the IG always showed a greater reduction in the preference scores than did the CG. Since 78.8% of participants had already completed ACP documents at T0, it can be assumed that they had already addressed their wishes and preferences. Therefore, changes over time may have been smaller than in a study population that had been less engaged with.

Overall, the results of this validation study indicate that the Treat-Me-ACP is appropriate for generating additional outcome parameters for the evaluation of ACP interventions and the dynamics of medical treatment preferences in ACP. Possible use cases are summarized in Additional file 4.

Given that average preference scores tended to decrease for eight of 10 preference scores in the CG, the instrument itself may have an effect on patients’ preferences. Study designs should therefore account for potential effects of scenarios on participants’ reflections about their wishes and preferences.

### Limitations

The study population is not representative for community-dwelling older persons in Germany. According to the German Federal Statistical Office, 60.2% of those in need of home care were female in 2019 [33], but in this study 78.5% of the patients are women. Women are therefore slightly overrepresented in this study. In addition, a large proportion of patients already had ACP documents at T0. Even though research on the prevalence of ACP documents in Germany is deficient, available data suggest that the prevalence of ACP documents in this study is well above average [34–37]. The study population therefore does not allow for generalization of the results. Furthermore, the study surveyed only a small sample. As a consequence, no statistically significant results were to be expected, and only trend statements about changes in patients’ preferences can be made. The small sample also did not allow further analysis of the sequence of scenarios and their impacts. Thus, no statements can be made about how response behavior may have been influenced by the scenario sequencing.

Additionally, the sample used for this validation study was not randomly selected. This raises the possibility of a double selection bias that could result from the patients’ decision to participate in the STADPLAN study and the selection by research assistants of participants for this sub-study based on patients’ condition.

The Treat-Me-ACP has not been validated or correlated with a gold standard. This should be carried out in further research to determine the content validity of the instrument.

## Conclusion

This study aimed to develop and validate the Treat-Me-ACP. The Treat-Me-ACP can be used to evaluate the dynamics of patients’ medical treatment preferences in ACP. In the international context, this study is one of few in recent years that has surveyed patient preferences in the ACP context based on scenarios. The instrument can be easily adapted for proxy assessment. During concurrent validation, we detected meaningful associations (1) between the scenario preference scores and the *how would you feel*-items and (2) between the preference scores and the global medical and home care goal.

### Electronic supplementary material

Below is the link to the electronic supplementary material.

Supplementary Material 1

Supplementary Material 2

Supplementary Material 3

Supplementary Material 4

## Data Availability

The datasets used and/or analysed during the current study are available from the corresponding author on reasonable request.

## Abbreviations

ACP: Advance Care Planning
CG: control group
c-RCT: cluster-randomized controlled trial
IG: intervention groupd
S: scenario
SPS: scenario preference score
STADPLAN: Study on Advance Care Planning in care-dependent community-dwelling older persons
TP: treatment preference
TPS: treatment preference scores
Treat-Me-ACP: Treatment-Preference-Measure-Advance Care Planning
