# Supplementary material for: Measuring patients’ medical treatment preferences in advance care planning: development and validation of the Treat-Me-ACP instrument – a secondary analysis of a cluster-randomized controlled trial
Source: BMC Palliat Care. 2024 Mar 21;23:77. doi: 10.1186/s12904-024-01404-8 (PMC10956243; doi:10.1186/s12904-024-01404-8)
Supplement: Supplementary file 1 — Supplementary Material 1 [file 12904_2024_1404_MOESM1_ESM.docx]

# Additional file 2: English translation of the German Treat-Me-ACP

Note: This English version of the Treat-Me-ACP is a non-validated English version of the Treat-Me-ACP to help understand the analyses of the main manuscript. The validation study was conducted with the German version of the Treat-Me-ACP.

## Treat-Me-ACP

Time: ____

### Global medical care goal-item

When you think about your future, which care/treatment do you think is better?

A care/treatment that allows you to live as long as possible. But this can cause health problems.

A care/treatment where your life is shorter. But you do not have major health problems. You are not burdened by it.

### Hypothetical scenarios

In the following, I describe various health conditions for you. Please answer the questions. And try to put yourself in these situations.

**Hypothetical scenario 1**: Current health status

S1 – *How would you feel-item*: You are in your current state of health. Your state of health is how you feel today. Imagine feeling that way for the rest of your life. What would that be like for you?

That kind of life would be all right for me.

That kind of life would be difficult. But I could handle it.

That kind of life would be barely livable or not livable at all.

S1.TP1: Imagine that you suddenly get a serious infection, such as pneumonia. If you take antibiotics, the infection can be cured. If you do not take antibiotics, you could get life-threatening complications or die. Would you want to take antibiotics?

Definitely not

Unlikely

Neither likely nor unlikely

Likely

Definitely

Not applicable

I do not want to answer

S1.TP2: Imagine your heart suddenly stopped beating. Would you like to be resuscitated?

Definitely not

Unlikely

Neither likely nor unlikely

Likely

Definitely

Not applicable

I do not want to answer

S1.TP3: Imagine you have an inflammation of the gallbladder. Surgical removal of the gallbladder can prevent life-threatening complications. This type of surgery is now considered routine, and the loss of the gallbladder can be tolerated without discomfort. Would you like to have your gallbladder removed?

Definitely not

Unlikely

Neither likely nor unlikely

Likely

Definitely

Not applicable

I do not want to answer

S1.TP4: Imagine you could no longer eat and drink by yourself. Would you wishant to be fed artificially for a foreseeable period of time?

Definitely not

Unlikely

Neither likely nor unlikely

Likely

Definitely

Not applicable

I do not want to answer

S1.TP5: Imagine you could no longer eat and drink by yourself. Would you want to be fed artificially for the rest of your life?

Definitely not

Unlikely

Neither likely nor unlikely

Likely

Definitely

Not applicable

I do not want to answer

**Hypothetical scenario 2:** Advanced dementia

Imagine you have very advanced dementia. This means

- You cannot think clearly. You are confused and often do not recognize your family members.
- You do not seem interested in what is going on in the world around you.
- You are not in pain. You are physically fine.
- There is no cure.

S2 – *How would you feel*-item: How would you feel if you spent the rest of your life in this state of health?

- That kind of life would be all right for me.
- That kind of life would be difficult. But I could handle it.
- That kind of life would be barely livable or not livable at all.

S2.TP1: Imagine that you suddenly get a serious infection, such as pneumonia. If you take antibiotics, the infection can be cured. If you do not take antibiotics, you could get life-threatening complications or die. Would you want to take antibiotics?

- Definitely not
- Unlikely
- Neither likely nor unlikely
- Likely
- Definitely
- Not applicable
- I do not want to answer

S2.TP2: Imagine your heart suddenly stopped beating. Would you like to be resuscitated?

- Definitely not
- Unlikely
- Neither likely nor unlikely
- Likely
- Definitely
- Not applicable
- I do not want to answer

S2.TP3: Imagine you have an inflammation of the gallbladder. Surgical removal of the gallbladder can prevent life-threatening complications. This type of surgery is now considered routine, and the loss of the gallbladder can be tolerated without discomfort. Would you like to have your gallbladder removed?

- Definitely not
- Unlikely
- Neither likely nor unlikely
- Likely
- Definitely
- Not applicable
- I do not want to answer

S2.TP4: Imagine you could no longer eat and drink by yourself. Would you wishant to be fed artificially for a foreseeable period of time?

- Definitely not
- Unlikely
- Neither likely nor unlikely
- Likely
- Definitely
- Not applicable
- I do not want to answer

S2.TP5: Imagine you could no longer eat and drink by yourself. Would you want to be fed artificially for the rest of your life?

- Definitely not
- Unlikely
- Neither likely nor unlikely
- Likely
- Definitely
- Not applicable
- I do not want to answer

**Hypothetical scenario 3:** Stroke with paralysis

Imagine you had a stroke.

- As a result, one side of your body is paralyzed. That is, one arm and one leg.
- You have difficulty expressing yourself clearly.
- You need help with all kinds of activities.

You have psychological damage from the stroke. Your doctor says the mental damage will not go away. Physically, however, you will recover from the event.

S3 – *How would you feel*-item: How would you feel if you spent the rest of your life in this state of health?

- That kind of life would be all right for me.
- That kind of life would be difficult. But I could handle it.
- That kind of life would be barely livable or not livable at all.

S3.TP1: Imagine that you suddenly get a serious infection, such as pneumonia. If you take antibiotics, the infection can be cured. If you do not take antibiotics, you could get life-threatening complications or die. Would you want to take antibiotics?

- Definitely not
- Unlikely
- Neither likely nor unlikely
- Likely
- Definitely
- Not applicable
- I do not want to answer

S3.TP2: Imagine your heart suddenly stopped beating. Would you like to be resuscitated?

- Definitely not
- Unlikely
- Neither likely nor unlikely
- Likely
- Definitely
- Not applicable
- I do not want to answer

S3.TP3: Imagine you have an inflammation of the gallbladder. Surgical removal of the gallbladder can prevent life-threatening complications. This type of surgery is now considered routine, and the loss of the gallbladder can be tolerated without discomfort. Would you like to have your gallbladder removed?

- Definitely not
- Unlikely
- Neither likely nor unlikely
- Likely
- Definitely
- Not applicable
- I do not want to answer

S3.TP4: Imagine you could no longer eat and drink by yourself. Would you wishant to be fed artificially for a foreseeable period of time?

- Definitely not
- Unlikely
- Neither likely nor unlikely
- Likely
- Definitely
- Not applicable
- I do not want to answer

S3.TP5: Imagine you could no longer eat and drink by yourself. Would you want to be fed artificially for the rest of your life?

- Definitely not
- Unlikely
- Neither likely nor unlikely
- Likely
- Definitely
- Not applicable
- I do not want to answer

**Hypothetical scenario 4:** Stroke with six weeks coma

Imagine you had a stroke.

- You were in a coma for six weeks.
- You need help with all activities.
- You can live in this condition for several years.

You have mental damage from the stroke. Your doctor says the mental damage will go away. Physically, however, you will not recover from the event.

S4 – *How would you feel*-item: How would you feel if you spent the rest of your life in this state of health?

- That kind of life would be all right for me.
- That kind of life would be difficult. But I could handle it.
- That kind of life would be barely livable or not livable at all.

S4.TP1: Imagine that you suddenly get a serious infection, such as pneumonia. If you take antibiotics, the infection can be cured. If you do not take antibiotics, you could get life-threatening complications or die. Would you want to take antibiotics?

- Definitely not
- Unlikely
- Neither likely nor unlikely
- Likely
- Definitely
- Not applicable
- I do not want to answer

S4.TP2: Imagine your heart suddenly stopped beating. Would you like to be resuscitated?

- Definitely not
- Unlikely
- Neither likely nor unlikely
- Likely
- Definitely
- Not applicable
- I do not want to answer

S4.TP3: Imagine you have an inflammation of the gallbladder. Surgical removal of the gallbladder can prevent life-threatening complications. This type of surgery is now considered routine, and the loss of the gallbladder can be tolerated without discomfort. Would you like to have your gallbladder removed?

- Definitely not
- Unlikely
- Neither likely nor unlikely
- Likely
- Definitely
- Not applicable
- I do not want to answer

S4.TP4: Imagine you could no longer eat and drink by yourself. Would you wishant to be fed artificially for a foreseeable period of time?

- Definitely not
- Unlikely
- Neither likely nor unlikely
- Likely
- Definitely
- Not applicable
- I do not want to answer

S4.TP5: Imagine you could no longer eat and drink by yourself. Would you want to be fed artificially for the rest of your life?

- Definitely not
- Unlikely
- Neither likely nor unlikely
- Likely
- Definitely
- Not applicable
- I do not want to answer

**Hypothetical scenario 5:** Incurable brain tumor

Imagine you have a malignant brain tumor.

- You feel tired and weak.
- You spend most of your time in bed.
- Your mental health is affected.
- You have no pain.

Your doctor says the cancer is incurable. He estimates that you have six months to live.

S5 – *How would you feel*-item: How would you feel if you spent the rest of your life in this state of health?

- That kind of life would be all right for me.
- That kind of life would be difficult. But I could handle it.
- That kind of life would be barely livable or not livable at all.

S5.TP1: Imagine that you suddenly get a serious infection, such as pneumonia. If you take antibiotics, the infection can be cured. If you do not take antibiotics, you could get life-threatening complications or die. Would you want to take antibiotics?

- Definitely not
- Unlikely
- Neither likely nor unlikely
- Likely
- Definitely
- Not applicable
- I do not want to answer

S5.TP2: Imagine your heart suddenly stopped beating. Would you like to be resuscitated?

- Definitely not
- Unlikely
- Neither likely nor unlikely
- Likely
- Definitely
- Not applicable
- I do not want to answer

S5.TP3: Imagine you have an inflammation of the gallbladder. Surgical removal of the gallbladder can prevent life-threatening complications. This type of surgery is now considered routine, and the loss of the gallbladder can be tolerated without discomfort. Would you like to have your gallbladder removed?

- Definitely not
- Unlikely
- Neither likely nor unlikely
- Likely
- Definitely
- Not applicable
- I do not want to answer

S5.TP4: Imagine you could no longer eat and drink by yourself. Would you wishant to be fed artificially for a foreseeable period of time?

- Definitely not
- Unlikely
- Neither likely nor unlikely
- Likely
- Definitely
- Not applicable
- I do not want to answer

S5.TP5: Imagine you could no longer eat and drink by yourself. Would you want to be fed artificially for the rest of your life?

- Definitely not
- Unlikely
- Neither likely nor unlikely
- Likely
- Definitely
- Not applicable
- I do not want to answer

Time: ____
