# Supplementary material for: Measuring patients’ medical treatment preferences in advance care planning: development and validation of the Treat-Me-ACP instrument – a secondary analysis of a cluster-randomized controlled trial
Source: BMC Palliat Care. 2024 Mar 21;23:77. doi: 10.1186/s12904-024-01404-8 (PMC10956243; doi:10.1186/s12904-024-01404-8)
Supplement: Supplementary file 2 — Supplementary Material 2 [file 12904_2024_1404_MOESM2_ESM.docx]

# Additional file 1: Development and pre-test of the Treat-Me-ACP

## Step 1: Development of a preliminary version

### Methods

- Translation of the Life Support Preferences Questionnaires [1, 2] and the Emanuel Medical Directive [3] into German using the TRAPD-approach [4]
- Rating according to relevance, acceptability and appropriateness for the German healthcare context. Elements that received consensus were used
- Translation into patient friendly language by experts for easy language

### Results:

- Instrument with nine scenarios:

1. Current health status
2. Advanced dementia
3. Shortness of breath due to coronary heart diseases or chronic obstructive pulmonary disease
4. Stroke with paralysis and speech disorder, with a certain probability that the condition will improve
5. Stroke with paralysis and speech disorder, with no prospect of recovery
6. Stroke with six weeks coma, with a certain probability that the condition will improve
7. Stroke with six weeks coma, with no prospect of recovery
8. Colorectal cancer with metastases in the liver, no pain
9. Colorectal cancer with metastases in the liver, daily use of painkiller
10. and one item to assess the patients’ global medical care goal

## Step 2: Assessment of comprehensibility, acceptance, and feasibility

### Methods

- 1^st^ round: ten cognitive interviews with probing questions with people without care need to optimize scenarios
- 2^nd^ round: five cognitive interviews with individuals with care needs to test wording and understandability and assess accepteance and feasibility

### Results

- Reduction to five scenarios where no ambiguity has occurred to reduce complexity and length of the instrument
- Replacement colorectal cancer by incurable brain tumor

## Step 3: Final interview round with adapted instrument

### Methods

- 13 interviews with probing questions to test for comprehensibility, acceptance, and feasibility again

### Results

- The final instrument consists of five scenarios

1. Current health status
2. Advanced dementia
3. Severe stroke with paralysis of one half of the body and speech disorder
4. Severe stroke with six weeks coma
5. Incurable brain tumor

and one item to assess the patients global medical care goal

References

1. Coppola KM, Bookwala J, Ditto PH, Lockhart LK, Danks JH, Smucker WD. Elderly adults' preferences for life-sustaining treatments: the role of impairment, prognosis, and pain. Death Stud. 1999;23:617–34. doi:10.1080/074811899200803.

2. Bookwala J, Coppola KM, Fagerlin A, Ditto PH, Danks JH, Smucker WD. Gender differences in older adults' preferences for life-sustaining medical treatments and end-of-life values. Death Stud. 2001;25:127–49. doi:10.1080/07481180126202.

3. Schwartz CE, Merriman MP, Reed GW, Hammes BJ. Measuring patient treatment preferences in end-of-life care research: applications for advance care planning interventions and response shift research. J Palliat Med. 2004;7:233–45. doi:10.1089/109662104773709350.

4. Harkness JA, van de Viljver FJR, Mohler PP, editors. Cross cultural survey methods. Hoboken, NJ: Wiley-Interscience; 2003.
