# Supplementary material for: Measuring patients’ medical treatment preferences in advance care planning: development and validation of the Treat-Me-ACP instrument – a secondary analysis of a cluster-randomized controlled trial
Source: BMC Palliat Care. 2024 Mar 21;23:77. doi: 10.1186/s12904-024-01404-8 (PMC10956243; doi:10.1186/s12904-024-01404-8)
Supplement: Supplementary file 3 — Supplementary Material 3 [file 12904_2024_1404_MOESM3_ESM.docx]

# Additional file 4: Use cases of the Treat-Me-ACP

Note: This document shows different examples of applications and interpretations of the Treat-Me-ACP instrument. These are some of the things we have thought about when developing the tool. To show the practical application of the Treat-Me-ACP, the mean value of the study population is used, but interpreted as belonging to one patient.

## Cross-sectional survey

### Make trend statements about whether life-sustaining treatments are more preferred or whether non-life-sustaining measures are more preferred

The *global medical goal*-item can be used to indicate a patient's (non)preference for life-sustaining treatment. This item consists of a dichotomous question in which the patient indicates whether he or she generally prefers care or treatment that will allow him or her to live as long as possible but may lead to health problems or whether he or she prefers a shorter life with major health problems.

The *global medical care goal*-item provides an opportunity for an initial assessment of preferences. In the next step, the preference scores can be used to check whether the global goal is reflected in the preference scores. If there is a preference for a shorter life with fewer health problems, the preference scores should be lower than if there is a preference for a longer life with possible health limitations.

### Identify current preferences for different health limitations (scenarios) and different invasive treatments (treatments)

The preference scores allow for a more detailed differentiation of preferences for different health states and treatments.

Figure s1 shows that the patient has the strongest preferences for life-sustaining interventions in the current state of health (scenario 1). Preferences for life-sustaining interventions are low for the remaining health states queried.

Figure s1: Scenario preference scores of the patient

Figure s2 shows that the patient has the strongest preferences for treatments that are likely to be associated with few long-term health limitations (antibiotics and cholecystectomy). Treatments that are more invasive or associated with more severe limitations are less preferred or not preferred at all.

Figure s2: Treatment preference scores of the patient

## Longitudinal survey

### Trend of preferences over time, in order to be able to make statements about the stability of preferences

Figures s3 and s4 show the patient's preference scores at two points in time. By plotting the two time points on a radar chart, the changes in preferences can be seen. This allows a statement to be made about the stability of the preferences. Additional time points can be used to determine the stability of preferences over time.

In the example shown, the curve for the second time point lies continuously within the curve for the first time point. This means that the preference for life-sustaining treatment has decreased slightly.

Figure s3: Scenario preference scores of the patient; comparison of two points in time

Figure s4: Treatment preference scores of the patient; comparison of two points in time

### Statements about changes in preferences after an intervention to analyse the effectiveness

By comparing preference scores at different time points, as shown in Figures s3 and s4, it is possible to make statements about changes in preference scores following an advance care planning intervention. The Treat-Me-ACP is therefore an instrument that can be used as a complementary tool to evaluate effectiveness. To assess effectiveness, the preference scores at different time points must be analyzed using appropriate statistical methods, which may differ depending on the study design.
