# Supplementary material for: Measuring patients’ medical treatment preferences in advance care planning: development and validation of the Treat-Me-ACP instrument – a secondary analysis of a cluster-randomized controlled trial
Source: BMC Palliat Care. 2024 Mar 21;23:77. doi: 10.1186/s12904-024-01404-8 (PMC10956243; doi:10.1186/s12904-024-01404-8)
Supplement: Supplementary file 4 — Supplementary Material 4 [file 12904_2024_1404_MOESM4_ESM.docx]

# Additional file 3: Missing values

Table s1: Missing values at baseline

| Item | Missing values (%) | SD |
| --- | --- | --- |
| Global medical and home care goal | 3.8 | 0.4 |
| S1 – Current health status | M: 3.5; MD: 2.5  IQR: 3.1 | 3.0 |
| S1 – *How would you feel*-item | 0.0 | 0.6 |
| S1.TP1 – Antibiotics | 1.3 | 1.1 |
| S1.TP2 – Resuscitation | 6.3 | 1.7 |
| S1.TP3 – Cholecystectomy | 8.8 | 1.2 |
| S1.TP4 – Temporary artificial nutrition | 2.5 | 1.7 |
| S1.TP5 – Permanent artificial nutrition | 2.5 | 1.1 |
| S2 – Advanced dementia | M: 5.2; MD: 4  IQR: 4 | 3.0 |
| S2 – *How would you feel*-item | 2.5 | 0.6 |
| S2.TP1 – Antibiotics | 2.5 | 1.7 |
| S2.TP2 – Resuscitation | 7.5 | 1.3 |
| S2.TP3 – Cholecystectomy | 10.0 | 1.7 |
| S2.TP4 – Temporary artificial nutrition | 3.8 | 1.5 |
| S2.TP5 – Permanent artificial nutrition | 5.0 | 0.9 |
| S3 – Stroke with paralysis | M: 5.6; MD: 5.0 IQR: 0.9 | 1.7 |
| S3 – *How would you feel-*item | 5.0 | 0.6 |
| S3.TP1 – Antibiotics | 5.0 | 1.6 |
| S3.TP2 – Resuscitation | 6.3 | 1.4 |
| S3.TP3 – Cholecystectomy | 8.8 | 1.6 |
| S3.TP4 – Temporary artificial nutrition | 3.8 | 1.5 |
| S3.TP5 – Permanent artificial nutrition | 5.0 | 0.9 |
| S4 – Stroke with six weeks coma | M: 7.1; MD: 6.9 IQR: 1.3 | 1.0 |
| S4 – *How would you feel*-item | 6.3 | 0.5 |
| S4.TP1 – Antibiotics | 7.5 | 1.6 |
| S4.TP2 – Resuscitation | 7.5 | 1.3 |
| S4.TP3 – Cholecystectomy | 8.8 | 1.5 |
| S4.TP4 – Temporary artificial nutrition d | 6.3 | 1.4 |
| S4.TP5 – Permanent artificial nutrition | 6.3 | 0.8 |
| S5 – Incurable brain tumor | M: 11.3; MD: 11.3  IQR: 1.9 | 1.8 |
| S5 – *How would you feel*-item | 8.8 | 0.5 |
| S5.TP1 – Antibiotics | 12.5 | 1.6 |
| S5.TP2 – Resuscitation | 11.3 | 1.2 |
| S5.TP3 – Cholecystectomy | 13.8 | 1.4 |
| S5.TP4 – Temporary artificial nutrition | 10.0 | 1.4 |
| S5.TP5 – Permanent artificial nutrition | 11.3 | 0.8 |
